# Performance of prediction rules and guidelines in detecting serious bacterial infections among Tanzanian febrile children

**DOI:** 10.1186/s12879-019-4371-y

**Published:** 2019-09-03

**Authors:** Kristina Keitel, Mary Kilowoko, Esther Kyungu, Blaise Genton, Valérie D’Acremont

**Affiliations:** 1 Swiss Tropical and Public Health Institute, University of Basel, Basel, Switzerland; 2 Department of Pediatric Emergency Medicine, University Hospital of Bern, Bern, Switzerland; 3 Ilala Municipality, Dar es Salaam, Tanzania; 4 Tanzanian Training Centre for International Health, Ifakara, Tanzania; 5 Center for Primary Care and Public Health (Unisanté), University of Lausanne, Lausanne, Switzerland; 6 Infectious Diseases Service, University Hospital Lausanne, Lausanne, Switzerland

**Keywords:** Clinical prediction rules, Serious bacterial infections, Childhood infections, External validation, Diagnostic accuracy, IMCI

## Abstract

**Background:**

Health-workers in developing countries rely on clinical algorithms, such as the Integrated Management of Childhood Illnesses (IMCI), for the management of patients, including diagnosis of serious bacterial infections (SBI). The diagnostic accuracy of IMCI in detecting children with SBI is unknown. Prediction rules and guidelines for SBI from well-resourced countries at outpatient level may help to improve current guidelines; however, their diagnostic performance has not been evaluated in resource-limited countries, where clinical conditions, access to care, and diagnostic capacity differ. The aim of this study was to estimate the diagnostic accuracy of existing prediction rules and clinical guidelines in identifying children with SBI in a cohort of febrile children attending outpatient health facilities in Tanzania.

**Methods:**

Structured literature review to identify available prediction rules and guidelines aimed at detecting SBI and retrospective, external validation on a dataset containing 1005 febrile Tanzanian children with acute infections. The reference standard, SBI, was established based on rigorous clinical and microbiological criteria.

**Results:**

Four prediction rules and five guidelines, including IMCI, could be validated. All examined rules and guidelines had insufficient diagnostic accuracy for ruling-in or ruling-out SBI with positive and negative likelihood ratios ranging from 1.04–1.87 to 0.47–0.92, respectively. IMCI had a sensitivity of 36.7% (95% CI 29.4–44.6%) at a specificity of 70.3% (67.1–73.4%). Rules that use a combination of clinical and laboratory testing had better performance compared to rules and guidelines using only clinical and or laboratory elements.

**Conclusions:**

Currently applied guidelines for managing children with febrile illness have insufficient diagnostic accuracy in detecting children with SBI. Revised clinical algorithms including simple point-of-care tests with improved accuracy for detecting SBI targeting in tropical resource-poor settings are needed. They should undergo careful external validation against clinical outcome before implementation, given the inherent limitations of gold standards for SBI.

**Electronic supplementary material:**

The online version of this article (10.1186/s12879-019-4371-y) contains supplementary material, which is available to authorized users.

## Background

Acute febrile illnesses are the most common presentation of young children attending outpatient settings worldwide [1]. Like in well-resourced settings, the majority of acute febrile illnesses are caused by viral pathogens requiring minimal supportive intervention; serious bacterial infections (SBI) have become increasingly rare with improving vaccination coverage and hygiene [2, 3]. However, the lack of adequate diagnostic tools makes it difficult to differentiate these viral diseases from the minority of children with SBI. Children with serious bacterial infections (SBI) often present with non-specific clinical signs and several concomitant symptoms [4]. Sub-standard management of children with infections has resulted in persistent high mortality from common childhood infections [5] and high-volume over-prescription of antibiotics [6].

Health workers rely on the World Health Organization (WHO) Integrated Management of Childhood Illnesses (IMCI) algorithm, which recommends presumptive treatment based on clinical signs and symptoms (besides the rapid diagnostic test for malaria that was introduced in the 2014 version, [7]). The Integrated Community Case Management guidelines (iCCM) is a simplified version of IMCI, geared towards community health workers [8, 9]. Due to the lack of available evidence when IMCI was initially developed, the algorithm was based mainly on expert opinion in addition to small derivation studies [10]. Though IMCI and iCCM have been implemented globally, their performance in detecting children with SBI has not been validated to date using stringent microbiological methods, instead of expert clinical diagnosis (and chest radiograph (CXR) in some studies) [11, 12]. Adherence to IMCI has been low. The reasons for nonadherence to IMCI are numerous and complex [13, 14], but one important aspect is the content: for example, IMCI lacks guidance in key areas, e.g. for patients with fever without clinical focus [7]. As a result, clinicians over-prescribe antibiotics out of the fear of missing patients with SBI [15]. Therefore, there is a need to improve current management guidelines for the primary care management of acute febrile illnesses, including evidence from economically developed countries. Here, a series of clinical and laboratory prediction rules and clinical guidelines, with different degrees of validation, have been designed for the management of febrile children in the ambulatory setting [16–27]. There is a growing body of evidence that the causes of acute non-malaria febrile illnesses in children in low- and high resource settings are in fact quite similar [2]: cosmopolitan viruses and bacteria are the causative agent in the vast majority of cases while tropical pathogens cause only a minority of febrile episodes at the outpatient level. Clinical signs and laboratory tests from such clinical prediction rules and guidelines developed in well-resourced countries may thus also be useful for detecting SBI in children in low-resource settings. However, external validation to support their use in resource-poor settings is lacking. This is especially important because of differences in clinical presentations (e.g. malaria co-infection), the health care system (e.g. access to care, the possibility of safety netting, the level of training of primary care providers).

## Methods

### Aim

The aim of this study was to estimate the diagnostic accuracy of existing prediction rules and clinical guidelines, including IMCI and iCCM, in identifying children with SBI in a resource-poor setting.

### Design

We performed an external, retrospective validation study of existing prediction rules and guidelines on a dataset collected prospectively in Tanzania that contains children aged 2 months to 10 years with fever presenting to outpatient care [2].

### Participants/ setting

The study population comprised 1005 children from a study on causes of fever in rural and urban Tanzania, the ‘Tanzanian Fever Study’ [2]. Briefly, children aged 2 months to 10 years with fever (axillary temperature of ≥38 °C) were enrolled consecutively at two outpatient clinics in 2008. Children with severe acute malnutrition and/or those requiring immediate live-saving procedures were excluded. This was partly for safety reasons, but also because WHO recommends antibiotic treatment for all febrile children with severe acute malnutrition as these patients have a distinct immune response putting them at high risk of SBI [7, 28]. All participants in the dataset, including children with malaria infection, were included into the validation exercise. We performed sensitivity analyses to assess the influence of malaria co-infection on the diagnostic performance (see below).

### Outcome definition

The outcome, SBI, i.e. a bacterial infection requiring antibiotic treatment, was defined as presence of one of the following: bacteremia (positive blood culture for a known pathogen), *Salmonella typhi* infection (positive blood-or stool culture, or positive specific IgM rapid diagnostic test), radiographic pneumonia, urinary tract infection (positive urine dipstick and urine culture), meningitis, bacterial gastroenteritis (positive stool culture), significant skin/soft tissue infections and other systemic bacterial infections not routinely detected by blood culture (rickettsiosis, coxiellosis, and leptospirosis). Definitions were based on the methodology used in the ‘Tanzania Fever Study’: for each patient, the final diagnosis (or diagnoses) was established with a computer-generated algorithm based on pre-defined clinical and microbiological criteria [2]. These criteria were derived from international guidelines as well as systematic reviews.

### Clinical and laboratory assessment

Investigators used standardized case report forms to record clinical findings, including 23 symptoms and their respective duration, potential travel history and/or sick contacts, known chronic conditions, and 49 clinical signs. At the initial visit a systematic set of investigations was performed according to predefined algorithm; malaria testing was done for all children [2]. If a clinical or laboratory diagnosis could not be made at the initial visit, a follow-up visit was scheduled for day 7 that included a full clinical and laboratory assessment for patients with persistent symptoms. In all cases, blood samples and pooled nasal and throat swabs were taken for microbiologic testing (cultures and rapid tests) on site and further serologic and molecular work-up in Switzerland and the USA. A complete blood cell count, including white blood cell count was done on site for all children. C-reactive protein (CRP) and procalcitonin (PCT) were performed retrospectively on stored samples by ELISA as detailed elsewhere [29]. CXR were performed in the subgroup of cases fulfilling the WHO clinical definition of pneumonia [30]. The diagnosis of radiological pneumonia was made in cases where CXR showed ‘primary endpoint consolidation’ according to WHO’s Pneumococcal Trialist Ad Hoc Committee recommendations [31]. If the IMCI clinical criteria for a suspected human immunodeficiency virus (HIV) infection were present, voluntary HIV testing was recommended to the child’s guardian.

### Selection of prediction rules and guidelines

All available prediction rules (laboratory and clinical) for identifying any SBI in children in the outpatient settings were identified through a structured literature review in Medline and Embase as part of the development of a novel disease management algorithm [32]. The search strategy is detailed in the Additional file 1 of the publication. The search was modified based on previously published systematic review and a European validation study [16, 33]. Prediction rules and guidelines that target the neonatal period, i.e. < 3 months, were excluded. We also did not include prediction rules that primarily aim at predicting death (such as the PEDIA [34], LODS [35], and SICK [36] scores) or the need for referral to the pediatric intensive care unit at in-patient level. Scores aimed at identifying dehydration for patients with gastroenteritis, or at detecting children with meningitis (there were only 2 patients with meningitis) were also not included. When variables of the dataset were not entirely matching the variables of the original rule or guideline, we identified proxies where possible (Additional file 2: Table S1). When more than 20% of the required variables were not recorded in the dataset (systematically missing), the rule/guideline was not included in the validation. This was based on the assumption that missing systematically more than 20% of predictor variables was not clinically sensible. Missing data on variables used in the validation were not imputed because the necessary missing-at-random assumption was likely to be incorrect given that all data was collected based on a predefined algorithm. We report the number of observations available for analysis of each prediction rule after application of the above assumptions. Where rules generated sum scores, previously published cut-offs were applied.

### Statistical analysis

We used the Standard for Reporting of Diagnostic Accuracy (STARD) guidelines for study reporting [37]. The accuracy of the included prediction rules and guidelines was estimated retrospectively in the prospectively collected ‘Tanzania Fever Study’ dataset by calculating sensitivity, specificity, and likelihood ratio (LR). For the low prevalence outpatient setting we considered a score helpful to rule-in SBI if, when positive, they substantially raised the probability of SBI (LR+ greater 5). Scores were deemed helpful for ruling-out SBI if, when negative, they substantially lowered the probability of illness (LR- lower than 0.2).

Clinical features were deemed warning signs if, when positive, they substantially raised the probability of illness—i.e., positive likelihood ratio of more than 5.0. Clinical features were deemed rule-out signs if, when negative, they substantially lowered the probability of illness—i.e., negative likelihood ratio of less than 0.2.

We performed the following sensitivity analyses by comparing the 95% confidence intervals (CIs) of diagnostic accuracy measures: First, to assess the influence of age range, we compared the target age group of the rules/ guidelines with those of the entire validation dataset. Second, as some predictors (fast breathing in IMCI, iCCM, and ALMANACH, and a positive CXR in the American Academy of Emergency Physicians [AAEP] guideline) were part of the diagnostic criteria for pneumonia in the validation dataset, we compared the full dataset with a dataset excluding pneumonia cases for these 4 guidelines. The same was done for UTI for prediction scales and guidelines that use urinary dipstick (Bleeker Score, Lab Score, ALMANACH and AAEP). Third, since malaria is known to raise CRP values [38], we compared malaria negative patients with the full dataset for prediction rules that contain CRP. Fourth, for prediction rules that were originally derived for children with fever without source, we compared the full dataset with the dataset containing children with fever without source only. All analyses were performed with Stata version 13.1. The confidence intervals were calculated using the Stata diagt procedure (http://www.stata.com/stb/stb59/sbe36_1/diagt.hlp. We used a web-based tool to generate Venn diagrams (http://jura.wi.mit.edu/bioc/tools/venn.php).

## Results

### Prediction rules and guidelines

**Fig. 1:**
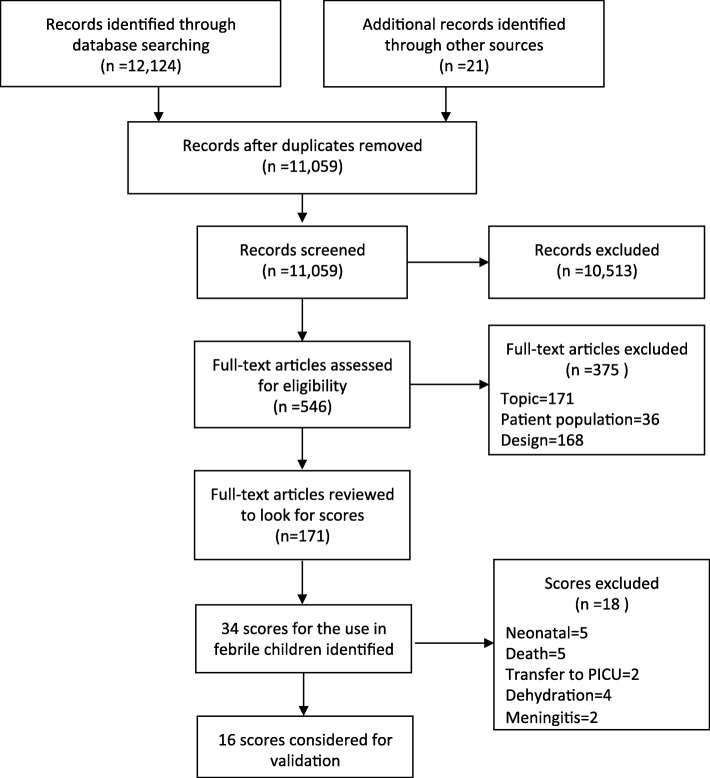
Flowchart of scores identified and considered for validation (adapted from [32]). Pediatric intensive care unit (PICU)

Through the structured literature review [32], we identified 34 prediction rules/guidelines for the use in febrile children. Sixteen were designed to predict SBI at the outpatient level (Fig. 1, Tables 1 and 2).

**Table 1:** Clinical and laboratory prediction rules for management of acute febrile illnesses in children^a^

Name of Prediction Rule	Age Group	Predictors											Derivation study
All serious infections													
Yale Observation Scale	0-24 m	Quality of Cry	Reaction to parents’ stimulation			State Variation		Color	Hydration	Response to social overtures			McCarthy et al. [39]
		Strong OR not crying(1)	Cries briefly (1)			Stays awake (1)		Pink (1)	Skin normal (1)	Smiles OR Alert (1)			
		Whimpering (3)	Cries on/off (3)			Awakes with stimulation (3)		Pale extremities (3)	Dry mouth (3)	Brief smile OR alerts briefly (3)			
		Weak (5)	Continual cry (5)			Falls to sleep (5)		Pale OR cyanotic (5)	Skin doughy (5)	No smile OR face anxious (5)			
		Sum of all six feature values (cut-offs used in literature: 8, 9 or 10)											
Five Stage Decision Tree	0–16 y	Clinician instinct that something is wrong		Dyspnea		Temperature > 39.95 °C			Diarrhea		Age 15-25 m		Van den Bruel et al. [17]
		No	0	No or unknown	0	No		0	No or unknown	0	No or unknown	0	
		Yes or unknown	1	Yes	1	Yes		1	Yes	1	Yes	1	
		If yes to any of these five features											
Bleeker	0-36 m	Duration fever Days (points)			H/o vomiting	Ill appearance	Chest wall retractions+ tachypnea	Poor peripheral circulation	WBC	CRP (mg/l)		Urine WBC	Bleeker et al. [18]
		0.5 (0), 1(2), 1.5 (4),2–2.5(5), 3–3.5(6), 4–4.5(7), 5–6(8), 6.5–8.5(9), ≥ 9(10)			Y = 5	Y = 4	Y = 12	Y = 7	< 10(0), 10–19(2), 20–29(4), 30–39(6), ≥ 40(8)	Divide value by 10 and round to lower integer, max. = 16 points		≥ 70 WBC/μl =9	
		Total points, described cutoffs: clinical: 10, lab: 8											
Thayyil	1-36 m	PCT (ng/ml)				CRP (mg/l)			WBC				Thayyil [19]
		> 2				> 50			> 15				
		Cutoff: All positive											
Lab Score	7d-36 m	PCT (ng/ml)				CRP (mg/l)			Urine Dipstick				Galetto-Lacour [20]
		< 0.5 (0), ≥0.5 (2), ≥2 (4)				< 40 (0), 40–99 (2), ≥100 (4)			Positive leucocyte or nitrite				
		Cutoff: 3											
AUS fever model	0–5 y	General appearance, cough, temperature, breathing difficulty, abnormal chest sounds, chronic disease, capillary refill time, urinary symptoms, respiratory rate, chest crackles, pneumococcal vaccine, heart rate, felt hot, meningococcal vaccine, infectious contacts, crying, fluid intake, respiratory symptoms, diarrhea, bulging fontanel, male, focal bacterial infection, abnormal ear, nose, and throat signs, age rash, stridor, wheeze											Craig et al. [21]
		Model risk estimate											
SBI risk score	1 m-15y	Developmental delay	Infection risk factor		State variation		T (°C)	CRT	Hydration		Tachypnea^b^	Hypoxia	Brent et al. [22]
		No (0) Yes (4)	No (0), Yes (2)		Eyes open (0) Eyes close briefly (1) Falls asleep (2)		< 37.5 (0), 37.5–38.3 (1) ≥38.4 (2)	< 2 (0) ≥2 (2)	Well hydrated (0) Dry mucous membranes (2) Reduced skin turgor (4)		No (0) Yes (1)	No (0) Mild (1) Severe (2)	
Rotterdam Fever model	1 m-16y	Age < 1, Sex, Duration of Fever, Height of Fever, Tachypnea, Tachycardia, SaO2 < 94%, CRT > 3 s, Chest Wall retraction, Ill-appearance, CRP											Nijman et al. [23]
		Model risk estimate											
Pneumonia													
Pneumonia Rule n°1	0–16 y	Parental concern illness is different						Shortness of breath					Van den Bruel et al. [17]
Values		If yes to any of these two features											
Pneumonia Rule n°2	0-21y	SaO2	Triage T		Wheeze		Decreased breath sounds		Focal rales	Chest pain	History of fever		Neuman et al. [24]
Values		Classification of Regression Tree/ clinical model											
Pneumonia Rule n°3	1-16y	Grunting			Cough		Rales		Decreased breath sound		Vomiting		Bilkis et al. [25]
		Model risk estimate											

*bpm* Beats per minute, *CRT* Capillary refill time, *CRP* C-reactive protein, *h/o* History of, *m* Months, *SaO2* Oxygen saturation, *PCT* Procalcitonin, *RR* Respiratory rate, *T* Body temperature, *Y* Yes, *y* Years, *WBC* White blood cell count

^a^Modified and appended from Verbakel et al. [33]

^b^Advanced pediatric life support cutoff

**Table 2:** Guidelines for management of acute febrile illnesses in children^a^

Name of guideline	Age Group	Clinical and laboratory features					Publication
IMCI	2 m-5y	CNS	Hydration/ nutrition		Respiratory	Other	
Danger signs		- Lethargic or unconscious -h/o convulsions or currently seizing -stiff neck	- Vomits everything -Unable to drink/ breastfeed -Severe malnutrition AND medical complications OR feeding issue -Severe dehydration (Two of the following) --Lethargic or unconscious --Sunken eyes --Not able to drink or drinking poorly --Reduced skin turgor		- Stridor in a calm child -SaO2 < 90% on RA (if available) -Chest indrawing and HIV positive	-Tender swelling behind ear -Severe palmar pallor -Severe complicated measles	WHO [7]
Indications for antibiotic treatment			-Uncomplicated severe malnutrition		-Cough and tachypnea and/or chest indrawing after trial of bronchodilator (2-12 m: RR > 50/min; ≥12 m: RR > 40/min)	-Ear pain or ear discharge < 14 days -Blood in stool	
		If yes to any of the danger signs: referral and IM antibiotics If yes to any of the antibiotic signs: oral antibiotic treatment					
iCCM	2 m-5y	CNS	Hydration/ nutrition		Respiratory	Other	
Danger signs		- Lethargic or unconscious -h/o convulsions or currently seizing	-Vomits everything -Unable to drink/ breastfeed -Severe malnutrition (low MUAC or bilateral edema)		-Chest indrawing	-HIV positive -Blood in stool	WHO [40]
Indications for antibiotic treatment					-Cough and tachypnea (2-12 m: RR > 50/min; ≥12 m: > 40/min)		
		If yes to any of the antibiotic signs; oral antibiotic treatment If yes to any of the danger signs: referral and oral antibiotics					
ALMANACH	2 m-5y	CNS	Hydration/ nutrition		Respiratory	Other	
Danger signs		- Lethargic or unconscious -h/o convulsions or currently seizing -stiff neck	-Vomits everything -Unable to drink/ breastfeed -Severe wasting -Severe dehydration (Two of the following) --Lethargic or unconscious --Sunken eyes --Not able to drink or drinking poorly --Reduced skin turgor		-Chest indrawing -Stridor in a calm child -Cyanosis	-Tender swelling behind ear -Severe pallor -Jaundice -Severe soft tissue infection	Rambaud-Althaus et al. [9]
Indications for antibiotic treatment					Cough and RR > 50/min	Acute ear discharge Blood in stool Urine dipstick (Positive leucocyte or nitrite) Abdominal tenderness	
		If yes to any of the antibiotic signs; oral antibiotic treatment If yes to any of the danger signs: referral and IM antibiotics					
NICE traffic light system	0-5y	Color	Activity	Respiratory	Circulation and Hydration	Other	NICE: Feverish Illness in Children [26]
Red- high risk		- Pale/mottled/ Ashen/blue	-No response to social cues -Appears ill to healthcare professional -Does not wake or if roused does not stay awake -Weak high-pitched or continuous cry	- Grunting - RR > 60/min - Moderate/severe chest indrawing	- Reduced skin turgor	- Age 0-3 m & T ≥ 38 °C - Non-blanching rash - Bulging fontanel - Neck stiffness - Status epilepticus - Focal neurological signs - Focal seizures	
Amber- intermediate risk		- Pallor	- Not responding normally to social cues - Wakes only with prolonged stimulation - Decreased activity - No smile	- Nasal flaring - Tachypnea (6-12 m: RR > 50/min; > 12 m: > 40/min) - SaO2 ≤ 95% - Crackles	-Tachycardia(< 12 m: > 160 bpm; 12-24 m: > 150 bpm; 2-5y: > 140 bpm)- Dry mucous membranes - Poor feeding in infants - CRT ≥ 3 s - Reduced urine output	- fever ≥5 days - swelling of a limb or joint - non-weight bearing limb/not using extremity - age 3-6 m, T ≥ 39 °C	
Values		If yes to any of these 5 categories, each scoring 2 to 13 features					
American Academy of Emergency Physicians Guidelines	3–36 m	Ill appearing	Positive chest radiography (to be obtained if:T ≥ 39 °Cand WBC > 20 K/mm3 or “clinical evidence of lower respiratory infection”	Positive urine leucocyte + nitrite (to be obtained in male < 1 year and female < 2 year)	T ≥ 39 °C And WBC > 15 K/mm3		American Academy of Emergency Physicians [27]
Values		If yes to any of these features					

*bpm* Beats per minute, *CRT* Capillary refill time, *CRP* C-reactive protein, *h/o* History of, *m* Months, *SaO2* Oxygen saturation, *PCT* Procalcitonin, *RR* Respiratory rate, *T* Body temperature, *Y* Yes, *y* Years, *WBC* White blood cell count

^a^Modified and appended from Verbakel et al. [33]

The NICE guideline is intended to predict ‘serious disease’ among children with acute febrile illness, and not to indicate antibiotic treatment. However, given that it was the only guideline designed for the use by healthcare professionals in primary care with various levels of training, we decided to include it in the validation exercise. In addition to the prediction rules and guidelines from the systematic review and European validation study [16, 33], we found one additional prediction rule for diagnosis of SBI [21], two prediction rules for pneumonia [24, 25], and four clinical guidelines (AAEP, IMCI, iCCM, and ALMANACH [7, 8, 27, 41]). ALMANACH is an improved IMCI-based algorithm that includes urinary dipstick testing [9]. Additional file 2: Table S1 displays whether the prediction rules and guidelines could be used for retrospective validation, as well as proxies for certain predictor variables used. For the prediction rules, validation was possible for the Bleeker Score, Thayyil Score, Lab Score and the Rotterdam Fever Model. More than 20% of predictors were missing systematically for other prediction rules, including 3 pneumonia rules. All clinical guidelines identified could be used for validation. Table 3 displays the prediction rules and guidelines that could be included into validation exercise. It also details the categories of SBI that were considered for the initial derivation or development of each rule/guideline.

**Table 3:** Prediction rules and guidelines that could be used for validation and SBI considered for each rule in the original derivation study/ at development

Prediction rule/guidelines	SBI categories considered									
	Meningitis	Pneumonia	Bacteremia	UTI	Typhoid	Cellulitis/ Abscess	Bacterial gastroenteritis	Leptospirosis	Intracellular bacteria	Other
Bleeker	✓	✓	✓	✓	✓^a^		✓			Osteomyelitis, Ethmoiditis
Thayyil	✓	✓	✓	✓	✓^a^					Any positive bacterial culture from an otherwise sterile site
Lab Score	✓	✓	✓	✓^b^	✓^a^	✓				
Rotterdam fever model^c^	✓	✓	✓	✓	✓^a^	✓	✓			Osteomyelitis
IMCI	✓	✓	✓	✓	✓	✓	✓	✓	✓	
iCCM	✓	✓	✓	✓	✓	✓	✓	✓	✓	
ALMANACH	✓	✓	✓	✓	✓	✓	✓	✓	✓	
NICE	✓	✓	✓	✓	✓	✓	✓			Osteomyelitis
AAEP	✓	✓	✓	✓	✓	✓	✓			Osteomyelitis

^a^Bacteremia only

^b^Pyelonephritis was defined as positive urine culture and positive DMSA scan

^c^Admission to the hospital was a pre-requisite for definition of SBI

### Validation dataset

The full details on the demographic and clinical characteristics of the study population are provided in the original study report [2]. A SBI was identified in 16% (162/1005) of patients in the validation dataset (Table 4).

**Table 4:** Cross table of serious bacterial infection (SBI) categories

	SBI categories, % (n), *N* = 1005								
	Meningitis	Pneumonia	Bacteremia	UTI	Typhoid	Cellulitis/ Abscess	Bacterial gastroenteritis	Leptospirosis	Intracellular bacteria
Meningitis	0.2 (2)	0	0	0	0	0	0	0	0
Pneumonia		3.1 (31)	0	0	0.4 (4)	0	0	0	0
Bacteremia^a^			1.7 (18)	0.4 (4)	0.4 (4)	0	0.1 (1)	0	0
UTI				5.9 (59)	0.1 (1)	0	0	0	0
Typhoid					3.7 (37)	0	0.2 (2)	0	0
Cellulitis/ Abscess						0.5 (5)	0	0	0
Bacterial gastroenteritis							1.4 (14)	0	0
Leptospirosis								0.4 (4)	0
Intracellular bacteria									1.1 (11)

^a^ positive blood culture for a known pathogen other than *Salmonella typhi*

### Validation results

The diagnostic accuracy for all included prediction rules and guidelines was low to moderate (Table 5). The Bleeker rule, Rotterdam Fever Model (2.5% risk cutoff), and NICE guidelines had the highest sensitivity, ranging from 77.3 to 83.7%. However, the specificity of the Bleeker score was only 40.8% (95% CI 36.9–44.9%), and those of the Rotterdam Fever Model (2.5% risk cutoff), and NICE guidelines even lower: 35.6% (95% CI 32.4–39.0%) and 25.2% (95% CI 22.6–28.6%), respectively. IMCI (like iCCM) had a very low sensitivity of 37.0% (95% CI 29.4–44.6%) and a moderate specificity of 70.3% (95% CI 67.1–73.4%). Compared to IMCI, ALMANACH had a higher sensitivity of 63.3% (55.4–70.6%). However, ALMANACH’s specificity was lower compared to IMCI (63.2, 95% CI 59.8–66.4%). None of the scores had LRs that would be considered helpful for ruling-in or ruling-out SBI in low-prevalence settings (LR+ greater 5 or LR- lower than 0.2).

**Table 5:** results of external validation of prediction rules and guidelines to rule-in and rule-out serious bacterial infection

Prediction rule/guideline	n/N^a^	%test positive	% sensitivity (95% CI)	% specificity (95% CI)	Likelihood ratio (95% CI)	
					positive	negative
Bleeker	126/731	62.9%	81.0 (73.0–87.4)	40.8 (36.9–44.9)	1.37 (1.23–1.52)	0.47 (0.32–0.68)
Thayyil	162/1001	5.2%	31.7 (24.7–39.4)	74.4 (71.3–77.4)	1.24 (0.96–1.60)	0.92 (0.82–1.03)
Lab Score	126/731	68.3%	70.6 (61.9–78.4)	32.2 (28.5–36.1)	1.04 (0.92–1.18)	0.91 (0.68–1.22)
Rotterdam fever model						
2.5% risk	161/985	66.50%	77.3 (70.1–83.5)	35.6 (32.4–39.0)	1.21 (1.10–1.32)	0.64 (0.47–0.86)
5% risk	161/985	55.53%	69.9 (62.3–76.9)	47.3 (43.9–50.8)	1.33 (1.18–1.50)	0.64 (0.50–0.81)
15% risk	161/985	36.24%	49.7 (41.8–57.6)	66.4 (63.1–69.6)	1.48 (1.23–1.78)	0.76 (0.65–0.89)
IMCI	164/1005	30.8%	36.7 (29.4–44.6)	70.3 (67.1–73.4)	1.22 (0.97–1.55)	0.90 (0.79–1.02)
iCCM	164/1005	30.5%	36.7 (29.4–44.6)	70.7 (67.5–73.7)	1.25 (1.00–1.57)	0.89 (0.79–1.01)
ALMANACH	164/1005	44.3%	63.3 (55.4–70.6)	63.2 (59.8–66.4)	1.72 (1.48–1.99)	0.58 (0.47–0.71)
NICE	164/1005	76.0%	83.7 (77.2–89.0)	25.5 (22.6–28.6)	1.12 (1.04–1.22)	0.64 (0.44–0.92)
AAEP	164/1005	41.7%	68.1 (60.4–75.1)	63.5 (60.2–66.8)	1.87 (1.63–2.14)	0.50 (0.40–0.63)

^a^Number of children with SBI out of all children included into validation. N represents the total number of children for which all variables of the prediction rule were recorded (please also refer to Additional file 2: Table S1)

**Fig. 2:**
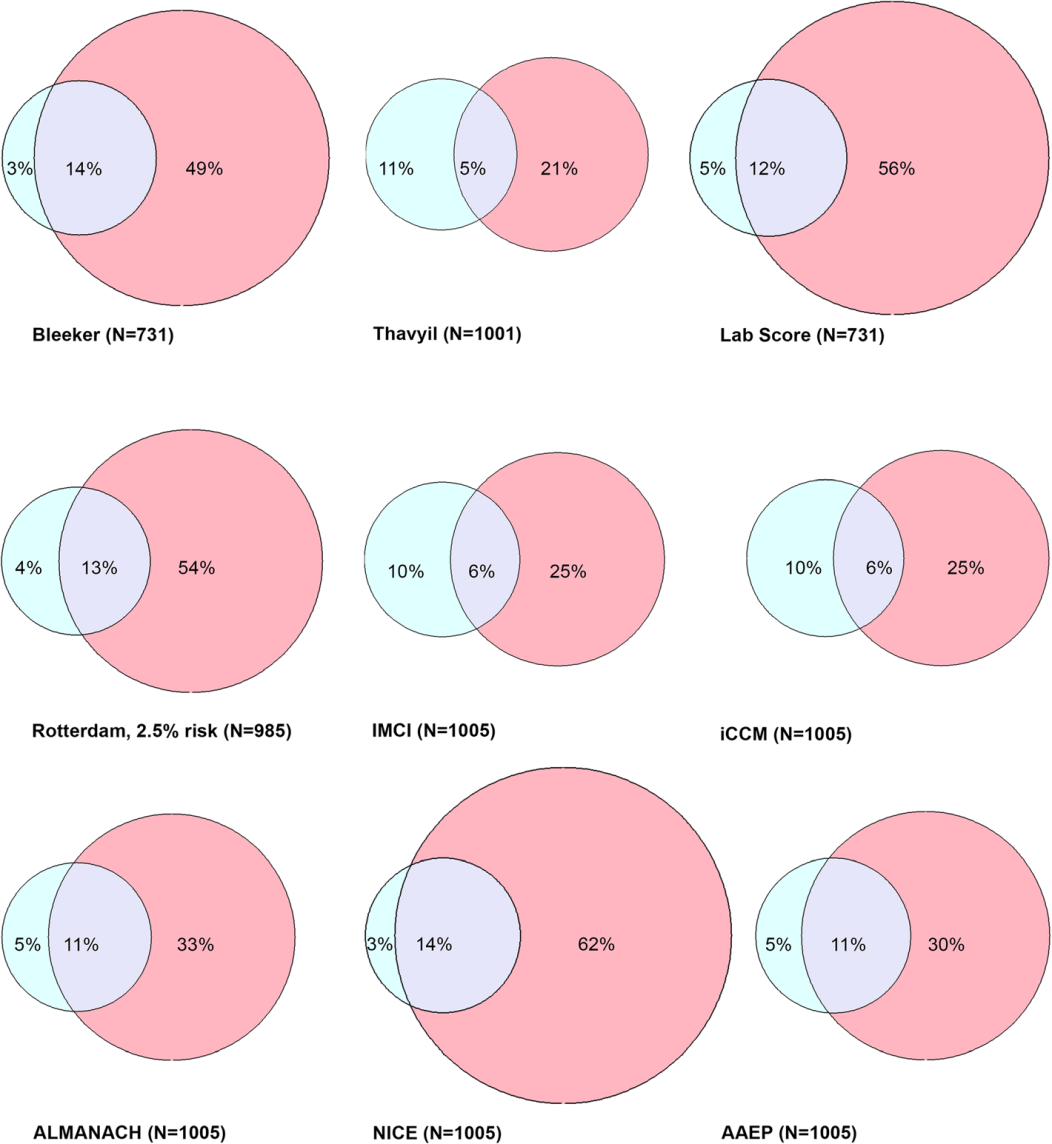
Overlap of serious bacterial infection classification (blue) and antibiotic treatment classification per rule or guideline (pink). The blue circles represent the percentage of patients with a SBI identified in the validation dataset. The pink circles illustrate the percentage of patients that tested ‘positive’ in the dataset per the rule or guideline. The overlap represents the percentage of patients with SBI who were correctly classified as such according to the rule

Figure 2 illustrates the overlap between SBI classification (reference) and antibiotic treatment classifications by the score. The Bleeker score and NICE guideline achieved the highest proportion of correct classifications (14% of the total population) but at the expense of many unnecessary antibiotic prescriptions: 49 and 62% of patients, respectively. IMCI, iCCM and the Thayyil score resulted in the lowest proportion of correct classifications (6% of patients).

**Fig. 3:**
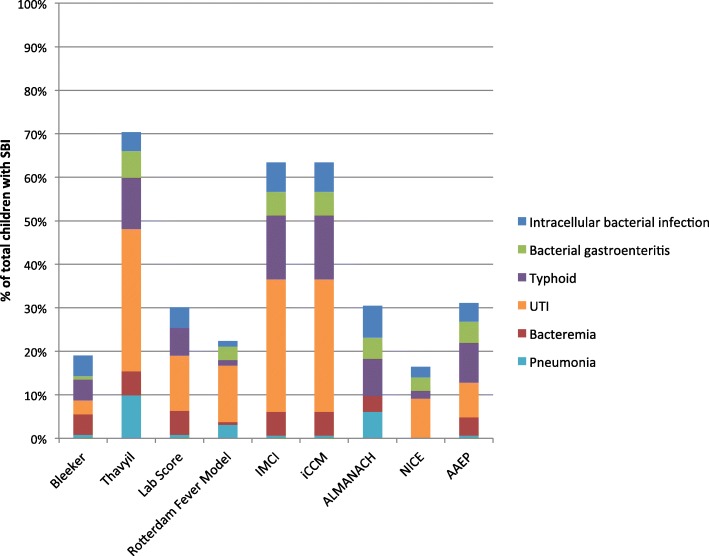
Missed cases of serious bacterial infections (SBI)

Figure 3 shows the missed cases of SBI according to different classifications. Not surprisingly, IMCI, iCCM, and AAEP missed very few pneumonia cases since the classifications used by these guidelines were part of the outcome definition (see Sensitivity analyses). Similarly, missed UTI cases were fewer in scores that use urine laboratory testing. All rules and guidelines, besides the Rotterdam model at low cutoff and the NICE guideline, missed a large amount of patients with bacteremia (50–75% of bacteremia cases).

### Sensitivity analyses

Applying the rule only to the age group for which it was originally designed, resulted in a significantly higher specificity for the Bleeker rule, Thayyil score, Lab Score and AAEP guideline (Table 6). We found similar results for relevant scores when including patients without pneumonia or without malaria only, when compared to the full validation dataset (Table 6). The specificity of ALMANACH was increased when applying to patients without UTI only. There was no significant change in the performance of prediction rules originally derived for children with fever without source when we compared the full dataset with the dataset containing children with fever without source only (Table 6).

**Table 6:** Results of sensitivity analyses

Prediction rule/guideline	n/N	%test positive	% sensitivity (95% CI)	% specificity (95% CI)	Likelihood ratio (95% CI)	
					positive	negative
Derivation age-group only						
Bleeker	88/507	46.5%	72.7 (62.2–81.7)	58.9 (54.1–63.7)	1.77 (1.49–2.10)	0.46 (0.33–0.66)
Thayyil	124/777	5.4%	11.3 (6.3–18.2)	95.7 (93.9–97.1)	2.63 (1.43–4.86)	0.93 (0.87–0.99)
Lab Score	88/507	54.2%	58.0 (47.0–68.4)	46.5 (41.7–51.4)	1.08 (0.89–1.32)	0.90 (0.69–1.18)
Rotterdam fever model						
2.5% risk	161/985	66.50%	77.6 (70.4–83.8)	35.7 (32.4–39.1)	1.21 (1.10–1.33)	0.63 (0.46–0.85)
5% risk	161/985	55.53%	70.2 (62.5–77.1)	47.3 (43.9–50.8)	1.33 (1.18–1.50)	0.63 (0.49–0.81)
15% risk	161/985	36.24%	49.7 (41.7–57.7)	66.4 (63.0–69.6)	1.48 (1.23–1.77)	0.76 (0.65–0.89)
IMCI	154/941	26.1%	31.8 (24.6–39.8)	70.0 (71.8–78.0)	1.27 (0.98–1.65)	0.91 (0.81–1.02)
iCCM	154/941	25.8%	31.8 (24.6–39.8)	75.3 (72.2–78.3)	1.29 (0.99–1.68)	0.90 (0.81–1.02)
ALMANACH	154/941	40.5%	64.9 (56.8–72.4)	64.3 (60.8–67.6)	1.82 (1.57–2.11)	0.55 (0.44–0.68)
NICE	154/941	74.4%	82.5 (75.5–88.1)	27.2 (24.1–30.4)	1.13 (1.04–1.23)	0.64 (0.45–0.93)
AAEP	122/756	22.5%	58.2 (48.9–67.1)	84.4 (81.3–87.1)	3.73 (2.95–4.72)	0.50 (0.40–0.61)
Patients without pneumonia only						
IMCI	133/974	28.7%	21.8 (15.1–29.8)	70.2 (66.9–73.2)	0.73 (0.52–1.02)	1.11 (1.01–1.23)
iCCM	133/974	28.4%	21.8 (15.1–29.8)	70.5 (67.3–73.6)	0.74 (0.53–1.04)	1.11 (1.00–1.23)
ALMANACH	133/974	43.5%	66.9 (58.2–74.8)	60.2 (56.8–63.5)	1.68 (1.45–1.94)	0.55 (0.43–0.70)
AAEP	133/841	39.9%	62.4 (53.6–70.7)	63.6 (60.3–66.9)	1.72 (1.46–2.01)	0.59 (0.47–0.74)
Patients without UTI only						
Bleeker	67/672	60.2%	70.1 (57.7–80.7)	40.8 (36.9–44.9)	1.19 (1.00–1.40)	0.73 (0.50–1.07)
Lab Score	67/672	67.9%	68.7 (56.2–79.4)	32.2 (28.5–36.1)	1.01 (0.85–1.20)	0.97(0.67–1.41)
ALMANACH	107/946	31.8%	48.6 (38.8–58.5)	70.3 (67.1–73.4)	1.64 (1.31–2.04)	0.73 (0.60–0.88)
AAEP	107/946	39.4%	62.6 (52.7–71.8)	63.5 (60.2–66.8)	1.72 (1.45–2.04)	0.59 (0.46–0.76)
Patients with negative malaria test only						
Bleeker	119/643	60.4%	79.8 (71.5–86.6)	43.9 (39.6–48.3)	1.42 (1.26–1.60)	0.46 (0.32–0.67)
Thayyil	153/897	24.0%	31.4 (24.1–39.4)	77.6 (74.4–80.5)	1.40 (1.07–1.83)	0.88 (0.79–0.99)
Lab Score	119/642	65.4%	68.9 (59.8–77.1)	35.4 (31.3–39.6)	1.07 (0.93–1.22)	0.88 (0.66–1.18)
Rotterdam fever model						
2.5% risk	152/881	62.9%	76.3 (68.7–82.8)	39.9 (36.3–43.6)	1.27 (1.14–1.41)	0.59 (0.44–0.80)
5% risk	152/881	51.1%	68.4 (60.4–75.7)	52.5 (48.8–56.2)	1.44 (1.26–1.65)	0.60 (0.47–0.77)
15% risk	152/881	32.1%	48.0 (39.9–56.3)	71.2 (67.8–74.5)	1.67 (1.36–2.04)	0.73 (0.62–0.86)
Patients with fever without source only for scores that were derived in children with fever without source						
Bleeker	73/315	69.8%	80.8 (69.9–89.1)	33.5 (27.6–39.8)	1.21 (1.05–1.40)	0.57 (0.35–0.95)
Thayyil	77/367	30.8%	27.3 (17.7–38.6)	68.3 (62.6–73.6)	0.86 (0.58–1.28)	1.07 (0.91–1.25)
Lab Score	73/314	72.2%	71.2 (59.4–81.2)	27.4 (21.9–33.5)	0.98 (0.83–1.16)	1.05 (0.69–1.59)

## Discussion

In the outpatient setting in Tanzania, none of the prediction rules and guidelines examined had sufficient diagnostic accuracy to detect children with SBI. IMCI and iCCM, which were designed to be sensitive for detecting SBI in these settings, actually had very low sensitivities when applied to our validation dataset. The Bleeker score, NICE guidelines, and Rotterdam Model at low cutoff showed the highest, though moderate, sensitivity, indicating a value in ruling-out children for SBI in low-prevalence, peripheral health care settings. However, at the same time, they classified many children as having a SBI, i.e. requiring antibiotic treatment. The use of such rules or guidelines would hence require further confirmatory testing to avoid antibiotic over-prescription. Rules that use a combination of clinical and laboratory testing, the Bleeker score, Rotterdam Model, ALMANACH, and AAEP guideline had better performance compared to rules and guidelines using only clinical and or laboratory elements. We performed several sensitivity analyses to estimate whether differences in demographic and ecological characteristics between the derivation and validation population had an influence on the diagnostic accuracy. Importantly, we did not find significant differences in the performance of the SBI scores in patients of the targeted age group or patients without malaria only when compared with the entire study population.

To our knowledge, this was the first comprehensive attempt to examine the accuracy of IMCI and other prediction rules and guidelines in diagnosing SBI in a tropical, low-resource outpatient setting against a robust gold standard. Besides one 1995 study in Bangladesh that performed blood cultures and CXR [12], guidelines developed for low-resource-settings (IMCI, iCCM, ALMANACH) have never been validated against carefully established gold standards (contrary to expert opinion). Overall guidance for SBI other than pneumonia and dysentery are lacking in the current IMCI guidelines, which specifies only “to give antibiotic treatment if a bacterial source of infection is identified”. But identifying such bacterial infections without guidance is challenging for low-level health workers. Alarmingly, the sensitivity of IMCI was very low—IMCI was originally designed to be very sensitive at the expense of being specific for detection of infections requiring antibiotic treatment. The diagnostic accuracy of ALMANACH sought to address these challenges through adding urinary dipstick testing and a clinical predictor for typhoid [41]. Indeed, sensitivity was improved but at the cost of a lower specificity in our dataset. Generally, very few studies have validated outpatient prediction rules and clinical guidelines for SBI systematically. One recent study validated systematically four clinical prediction rules and two national guidelines retrospectively across datasets from primary care and emergency departments in Europe [33]. The diagnostic accuracy of the prediction rules and guidelines also validated in our study were generally higher. This may be due to the fact that the original derivation population was more similar to the validation datasets of the European validation study. Other studies in the African setting have evaluated scores for SBI and death at the inpatient level. Nadjm et al. evaluated prospectively the accuracy of WHO hospital-level clinical criteria for presumptive antibiotic treatment in detecting SBI (positive blood and/or cerebrospinal fluid culture) among 3639 admitted children in Tanzania [42]. The sensitivity was higher when compared to IMCI in our study (67.4, 95% CI 65.9–69.0%), at a lower specificity of 51.5% (95% CI 49.9–53.1%). Reported sensitivities of a similar study by Berkley at al. were even higher [43]. However, the comparison of results from these studies with the present analysis is extremely limited by the difference in prevalence of SBI in the inpatient versus outpatient setting, and the restricted number of investigations for SBI performed (blood and cerebrospinal fluid culture only). Conroy et al. validated three scores to predict in-hospital (and not outpatient) mortality among Ugandan children with fever [44]. Through mortality is a relevant and robust outcome, its use at the outpatient level, where death is a rare event, is difficult.

This study has several limitations. Only a single dataset from was available for validation, which limits the generalizability of our findings. However, rates of bacteremia in our study were similar to other studies conducted at primary care level around the same time and the dataset is likely representative of the typical case-mix [45]. There are multiple sources of heterogeneity. The most obvious one is the difference in setting for all prediction rules and two out of the four guidelines. Difference in bacterial pathogens, such as typhoid and rickettsial diseases, substantially limits the applicability of “Northern” guidelines to tropical settings. Differences in recorded values between the derivation and validation datasets is another limitation for this analysis. Though this study used robust, predefined reference criteria with extensive microbiological testing, the gold standards for SBI certainly remain imperfect [46]. For pneumonia end-point consolidation on CXR has been used though it is known that only an (unknown) percentage of consolidations are of bacterial origin, and that viral pneumonia may produce abnormalities on CXR as well [47]. As a result, test diagnostic accuracy may be biased in both directions. The diagnostic accuracy of all available tests for typhoid is poor [48] and hence the typhoid classification (combination of rapid test and blood and stool cultures), was certainly suboptimal. Consequently, the sensitivity of guidelines to detect SBI may have been underestimated. Despite the comprehensive set of clinical and laboratory predictors in the validation dataset, we were able to validate only four of the nine prediction rules plus all guidelines and had to use proxies for several predictors. For the Bleeker score, for example, “ill-appearance” was likely underestimated in our validation dataset since the variables “lethargy, and very sick child” refer to a sicker child. On the other hand, using the urine leucocyte dipstick test instead of the urine WBC likely overestimated the presence of UTI. We did not impute missing data as the “missing at random assumption” could not be assumed for the validation dataset; this may have influenced our estimates of performance for those rules that use urinary dipstick testing where we encountered a large percentage of missing data in the validation set.

Our findings have several implications for clinical practice and research in low-resource settings. First, the efforts should be made to increase the sensitivity of current screening tools for SBI. As it was intended for IMCI, clinical guidelines should have high sensitivity as the access to care in such settings is difficult, referral to higher level of care may be delayed, and safety-netting is not always available. Guidelines should be presented as stepwise decision algorithms, which follow the logical flow of the actual diagnostic process [46]. This is especially true for low-resource settings where health care providers with limited training benefit from clinical decision algorithms [49]. Within such algorithms, simple but sensitive clinical criteria will be needed to quickly rule-out children with SBI. This could then be followed by a more specific second-step laboratory testing, such as point-of-care biomarkers, in order to avoid unnecessary antibiotic treatment. However, no algorithm will have perfect diagnostic accuracy making safety netting (follow-up) an important component of clinical care. Third, disease management algorithms should undergo careful external validation before implementation. Ideally, such validation studies should be performed against clinical outcome, and not against a microbiological reference standard only as it is difficult to establish a valid microbiological reference standard. This could either be achieved through composite reference standards including clinical patient follow-up [46], or through the evaluation of decision rules through randomized clinical trials [32].

Viral infections, such as bronchiolitis, may cause severe disease. The guidance on supportive measures for viral infections by a clinical algorithm designed for the low-resource outpatient setting may be become equally important with declining prevalence of SBI. ALMANACH, for example, achieved better clinical outcome in a validation study against routine care in Tanzania [50].

## Conclusions

None of the examined prediction rules and guidelines had sufficient diagnostic accuracy to detect children with SBI in a tropical, low-resource setting. IMCI and iCCM, which were designed to be sensitive for detecting SBI in these settings, actually had very low sensitivities when applied to our validation dataset. Some prediction rules and guidelines had higher sensitivity and hence showed promise to rule-out SBI in our dataset. However, they also classified a larger number of patients as having a SBI, calling for additional second-stage testing, such as point-of care inflammatory markers, and tests for severity such as oximetry and hemoglobin. New clinical algorithms should undergo careful external validation studies against clinical outcome before implementation in routine care.

## Additional files

Additional file 1: Literature search terms. (DOCX 126 kb)

Additional file 2: **Table S1.** Prediction rules and guidelines used for validation and proxy variables (if applicable). (PDF 58 kb)

## Data Availability

The dataset used for this analysis is available online as detailed in the original manuscript [3].

## Abbreviations

AAEP: American Academy of Emergency Physicians
CI: Confidence interval
CRP: C-reactive protein
CXR: Chest radiograph
HIV: Human immunodeficiency virus
iCCM: Integrated Community Case Management guidelines
IMCI: Integrated Management of Childhood Illnesses
LR: Likelihood ratio
SBI: Serious bacterial infection
UTI: Urinary tract infection
WHO: World Health Organization
