# Supplementary material for: Performance of prediction rules and guidelines in detecting serious bacterial infections among Tanzanian febrile children
Source: BMC Infect Dis. 2019 Sep 3;19:769. doi: 10.1186/s12879-019-4371-y (PMC6724300; doi:10.1186/s12879-019-4371-y)
Supplement: Supplementary file 1 — Literature search terms. (DOCX 126 kb) [file 12879_2019_4371_MOESM1_ESM.docx]

| **Literature search terms** | | |
| --- | --- | --- |
| **Search** | **PubMED** | **Embase** |
| Severe infections/ serious bacterial infections | ("Community-Acquired Infections"[Mesh] OR "Sepsis"[Mesh:NoExp] OR "Bacterial Infections"[Mesh] OR "Respiratory Tract Infections"[Mesh] OR "Shock, Septic"[Mesh] OR "Status Asthmaticus"[Mesh] OR "Meningitis"[Mesh:NoExp] OR "Meningitis, Bacterial"[Mesh] OR "Arthritis, Infectious"[Mesh] OR "Bone Diseases, Infectious"[Mesh] OR "Cellulitis"[Mesh] OR "Skin Diseases, Bacterial"[Mesh:NoExp] OR "Skin Diseases, Infectious"[Mesh:NoExp] OR "Ecthyma"[Mesh] OR "Erysipelas"[Mesh] OR "Staphylococcal Skin Infections"[Mesh] OR "Soft Tissue Infections"[Mesh] OR "Diarrhea, Infantile"[Mesh] OR "Dysentery"[Mesh] OR "Urinary Tract Infections"[Mesh] OR "Pyelonephritis"[Mesh:NoExp] OR "Typhoid Fever"[Mesh] OR "Fever of Unknown Origin"[Mesh] OR bacterial infection*[tiab] OR serious infection*[tiab] OR severe infection*[tiab] OR invasive infection*[tiab] OR (death[tiab] AND infection[tiab]) OR severe malaria[tiab] OR severe bronchiolitis[tiab] OR sepsis[tiab] OR meningitis[tiab] OR dehydration[tiab] OR pneumonia[tiab] OR pyelonephritis[tiab] OR urinary tract infection*[tiab] OR typhoid[tiab] OR leptospirosis[tiab] OR rickettsia*[tiab] OR osteomyelitis[tiab] OR cellulitis[tiab] OR bacteremia[tiab] OR bacteraemia[tiab] OR otitis media[tiab]) AND ("Signs and Symptoms"[Mesh:NoExp] OR "Seizures"[Mesh] OR "Abdominal Pain"[Mesh] OR "Vomiting"[Mesh] OR "Pallor"[Mesh] OR "Jaundice"[Mesh] OR "Tachypnea"[Mesh:NoExp] OR "Respiratory Sounds"[Mesh:NoExp] OR "Dyspnea"[Mesh:NoExp] OR "Vital Signs"[Mesh] OR "Tachycardia"[Mesh:NoExp] OR "Diagnostic Tests, Routine"[Mesh] OR "Physical Examination"[Mesh:NoExp] OR "Diagnosis"[Mesh:NoExp] OR "Clinical Decision-Making"[Mesh] OR "Medical History Taking"[Mesh:NoExp] OR "Symptom Assessment"[Mesh] OR "Odds Ratio"[Mesh] OR "Sensitivity and Specificity"[Mesh:NoExp] OR "Predictive Value of Tests"[Mesh] OR "ROC Curve"[Mesh] OR "Severity of Illness Index"[Mesh] OR "Decision Trees"[Mesh] OR "C-Reactive Protein"[Mesh] OR "Anemia"[Mesh:NoExp] OR clinical sign*[tiab] OR clinical syndrome* [tiab] OR "syndromic diagnosis"[tiab] OR clinical variable*[tiab] OR clinical predictor*[tiab] OR vital sign*[tiab] OR clinical feature*[tiab] OR "signs and symptoms"[tiab] OR red flag*[tiab] OR danger sign*[tiab] OR "abnormal mental status"[tiab] OR "altered mental status"[tiab] OR convulsion*[tiab] OR "stiff neck"[tiab] OR meningeal sign*[tiab] OR prostration[tiab] OR "chest wall retraction"[tiab] OR "chest indrawing"[tiab] OR stridor[tiab] OR tachypnea[tiab] OR "fast breathing"[tiab] OR tachypnoea[tiab] OR "respiratory rate"[tiab] OR tachycardia[tiab] OR "fast heart rate"[tiab] OR "capillary refill time"[tiab] OR vomiting[tiab] OR pallor[tiab] OR fever[tiab] OR algorithm*[tiab] OR decision tree*[tiab] OR prediction rule*[tiab] OR imci[tiab] OR "integrated management of childhood illness"[tiab] OR "severe anemia"[tiab] OR "severe anaemia"[tiab] OR procalcitonin[tiab] OR "C-reactive protein"[tiab] OR "Urine dipstick"[tiab] OR Urine leucocyte*[tiab] OR Urine nitrite*[tiab] OR "diagnostic accuracy"[tiab]) AND ("infant"[Mesh] OR "Child, Preschool"[Mesh] OR infant*[tiab] OR child*[tiab] pediatric*[tiab] OR paediatric*[tiab] OR under-five*[tiab] OR 59 month*[tiab] OR under-5[tiab] OR babies[tiab] OR baby[tiab]) | ('bacterial infection'/de OR Infection/de OR 'Enterobacteriaceae infection'/exp OR 'Gram negative infection'/exp OR 'Gram positive infection'/exp OR 'pyonephrosis'/exp OR 'rickettsiosis'/exp OR 'Staphylococcus infection'/exp OR 'Streptococcus infection'/exp OR 'community acquired infection'/exp OR 'sepsis'/de OR 'bacteremia'/de OR 'septic shock'/de OR 'septicemia'/de OR 'respiratory tract infection'/exp OR 'pneumonia'/de 'bronchopneumonia'/exp OR 'asthmatic state'/exp OR 'meningitis'/de OR 'bacterial meningitis'/exp OR 'Haemophilus meningitis'/exp OR 'pneumococcal meningitis'/exp OR 'bone infection'/de OR 'bacterial arthritis'/exp OR 'soft tissue infection'/exp OR 'cellulitis'/exp OR 'bacterial skin disease'/de 'erysipelas'/de OR 'impetigo'/exp OR 'skin abscess'/exp OR 'staphylococcal skin infection'/exp OR 'dysentery'/exp OR 'urinary tract infection'/exp OR 'acute pyelonephritis'/exp OR 'urinary tract infection'/exp OR 'typhoid fever'/exp OR ((bacterial NEXT/1 infection*) OR (serious NEXT/1 infection*) OR (severe NEXT/1 infection*) OR (invasive NEXT/1 infection*) OR (severe NEXT/1 malaria) OR (severe NEXT/1 bronchiolitis) OR sepsis OR meningitis OR pneumonia OR pyelonephritis OR (urinary NEXT/1 tract NEXT/1 infection*) OR typhoid OR leptospirosis OR rickettsia* OR osteomyelitis OR cellulitis OR bacteremia OR bacteraemia OR (otitis NEXT/1 media)):ab,ti) OR ('pallor'/exp OR 'seizure'/de OR 'convulsion'/de OR 'abdominal pain'/de OR 'vomiting'/de OR 'jaundice'/de OR 'abnormal respiratory sound'/exp OR 'dyspnea'/de OR 'tachypnea'/de OR 'vital sign'/exp OR 'heart rate'/de OR 'breathing rate'/de OR 'oxygen saturation'/exp OR 'tachycardia'/de OR 'diagnostic accuracy'/de OR 'diagnostic test accuracy study'/de OR 'classification algorithm'/exp OR 'practice guideline'/de OR 'predictive value'/exp OR 'diagnostic value'/de OR 'diagnostic accuracy'/de OR 'C reactive protein'/exp OR 'procalcitonin'/exp OR 'anemia'/de OR ((clinical NEXT/1 sign*) OR (clinical NEXT/1 syndrome*) OR (clinical NEXT/1 variable*) OR (clinical NEXT/1 predictor*) OR (vital NEXT/1 sign*) OR (clinical NEXT/1 feature*) OR "signs and symptoms" OR (red NEXT/1 flag*) OR (danger NEXT/1 sign*) OR "abnormal mental status" OR "altered mental status" OR convulsion* OR "stiff neck" OR (meningeal NEXT/1 sign*) OR prostration OR "chest wall retraction" OR "chest indrawing" OR stridor OR tachypn*a OR "fast breathing" OR "respiratory rate" OR tachycardia OR "fast heart rate" OR "capillary refill time" OR vomiting OR pallor OR fever OR algorithm* OR (decision NEXT/1 tree*) OR (prediction NEXT/1 rule*) OR imci OR "integrated management of childhood illness" OR "severe an*emia" OR procalcitonin OR "C-reactive protein" OR "Urine dipstick" OR (Urine NEXT/1 leucocyte*) OR (Urine NEXT/1 nitrite*) OR "diagnostic accuracy"):ab,ti) |
| Dehydration | (dehydration[tiab] OR "Dehydration"[Mesh]) AND ("Signs and Symptoms"[Mesh:NoExp] OR "Vital Signs"[Mesh] OR "Tachycardia"[Mesh:NoExp] OR "Diagnostic Tests, Routine"[Mesh] OR "Physical Examination"[Mesh:NoExp] OR "Diagnosis"[Mesh:NoExp] OR "Odds Ratio"[Mesh] OR "Sensitivity and Specificity"[Mesh:NoExp] OR "Predictive Value of Tests"[Mesh] OR "ROC Curve"[Mesh] OR "Severity of Illness Index"[Mesh] OR "Decision Trees"[Mesh] OR clinical sign*[tiab] OR clinical predictor* [tiab] OR vital sign* [tiab] OR clinical feature* [tiab] OR "signs and symptoms"[tiab] OR algorithm* [tiab] OR decision tree* [tiab] OR prediction rule* [tiab] OR imci [tiab] OR "integrated management of childhood illness"[tiab] OR "diagnostic accuracy"[tiab]) AND (infant*[tiab] OR child* [tiab] pediatric*[tiab] OR paediatric*[tiab] OR under-five* [tiab] OR 59 month* [tiab] OR under-5 [tiab] OR babies[tiab] OR baby[tiab] OR "infant"[Mesh] OR "Child, Preschool"[Mesh]) | (dehydration:ti,ab OR 'dehydration'/exp) AND ((clinical sign* OR clinical syndrome* OR clinical predictor* OR vital sign* OR clinical feature* OR "signs and symptoms" OR algorithm* OR decision tree* OR prediction rule* OR imci OR "integrated management of childhood illness" OR "diagnostic accuracy"):ti,ab OR 'vital sign'/exp OR 'heart rate'/de OR 'tachycardia'/de OR 'diagnostic accuracy'/de OR 'diagnostic test accuracy study'/de OR 'classification algorithm'/exp OR 'practice guideline'/de OR 'predictive value'/exp OR 'diagnostic value'/de OR 'symptom assessment'/de OR 'diagnostic accuracy'/de) AND ((infant* OR child* pediatric* OR paediatric* OR under-five* OR 59 month* OR under-5 OR babies OR baby):ti,ab OR 'preschool child'/exp OR 'infant'/exp) |
| Malnutrition | (malnutrition[tiab] OR "Malnutrition"[Mesh]) AND ("Anthropometry"[Mesh:NoExp] OR "Body Weights and Measures” [Mesh] OR weight*[tiab] OR *height[tiab] OR *length[tiab] OR MUAC[tiab] OR “mid upper arm circumference”[tiab]) AND (infant*[tiab] OR child* [tiab] pediatric*[tiab] OR paediatric*[tiab] OR under-five* [tiab] OR 59 month* [tiab] OR under-5 [tiab] OR babies[tiab] OR baby[tiab] OR "infant"[Mesh] OR "Child, Preschool"[Mesh]) | (malnutrition:ti,ab OR 'malnutrition'/exp) AND ('anthropometric parameters'/exp OR (weight* OR length OR height OR muac OR “mid upper arm circumference”):ti,ab) AND ((infant* OR child* pediatric* OR paediatric* OR under-five* OR 59 month* OR under-5 OR babies OR baby):ti,ab OR 'preschool child'/exp OR 'infant'/exp) |
