# Supplementary material for: Performance of prediction rules and guidelines in detecting serious bacterial infections among Tanzanian febrile children
Source: BMC Infect Dis. 2019 Sep 3;19:769. doi: 10.1186/s12879-019-4371-y (PMC6724300; doi:10.1186/s12879-019-4371-y)
Supplement: Supplementary file 2 — Table S1. Prediction rules and guidelines used for validation and proxy variables (if applicable). (PDF 58 kb) [file 12879_2019_4371_MOESM2_ESM.pdf]

Supplementary Table 1: Prediction rules and guidelines used for validation and proxy variables (if applicable)

Prediction Rule	used for validation	% of predictors	variables used (original or proxy)							
**All serious bacterial infections**										
**Yale Observation Scale**			Quality of Cry	Reaction to parent stimulation	State Variation	Colour	Hydration	Response to social overtures		
	NO	17%	NO	NO	NO	NO	YES	NO		
**5-stage Decision Tree**			Something is wrong	Dyspnoea	Temperature	Diarrhoea	Age			
	NO	60%	NO	NO	YES	YES	YES			
**Bleeker**			Duration fever	Vomiting	Ill appearance	Chest wall retractions	Poor peripheral circulation	WBC	CRP	Urine WBC
	YES	88%	YES	YES	(YES)	YES	NO	YES	YES	(YES)
% n/N			98.0%	100.0%	100.0%	99.7%	0.0%	99.8%	100.0%	68.6%
			duration of fever	vomiting	"drowsy", "lethargic", "very sick child"	lower chest indrawing				urine leukocytes*
**Thavyil**			PCT	CRP	WBC					
	YES	100%	YES	YES	YES					
% n/N			99.8%	100.0%	98.0%					
**Lab Score**			PCT	CRP	Urine Dipstick					
	YES	100%	YES	YES	YES					
% n/N			99.8%	100.0%	68.5% *					
**AUS fever model**			General appearance, cough, temperature, breathing difficulty, abnormal chest sounds, chronic disease,capillary refill time, urinary symptoms, respiratory rate, chest crackles, pneumococcal vaccine, heart rate, felt hot, meningococcal vaccine, infectious contacts, crying, fluid intake, respiratory symptoms, diarrhea, bulging fontanelle, male, focal bacterial infection, abnormal ear/nose/throat signs, age, rash, stridor, wheeze							
	NO	67%	18/27							
**SBI risk score**			Developmental delay	Infection risk factor	State variation	Temperature	Capillary Refill Time	Hydration	Tachypnea	Hypoxia
	NO	63%	YES	YES	NO	YES	NO	(YES)	YES	NO

Prediction Rule	used for validation	% of predictors	variables used (original or proxy)										
**Rotterdam fever model**			Age	Sex	Duration of Fever	Height of Fever	Tachypnea	Tachycardia	Hypoxia	Capillary Refill Time	chest wall indrawing	ill-appearance	CRP
	YES	82%	YES	YES	YES	YES	YES	YES	NO	NO	YES	(YES)	YES
% n/N			100.0%	100.0%	98.0%	100.0%	99.7%	98.7%	0.0%	0.0%	100.0%	100.0%	100.0%
												"drowsy", "lethargic", "very sick	
**Pneumonia Rule n°1**			Illness is different (parent)	Dyspnea									
	NO	50%	NO	NO									
**Pneumonia Rule n°2**			Oxygen saturation	Temperature	Wheeze	Decreased breath sounds	Focal rales	Chest pain	History of fever				
	NO	57%	NO	YES	YES	NO	NO	YES	YES				
**Pneumonia Rule n°3**			Grunting	Cough	Focal rales	Decreased breath sounds	Vomiting						
	NO	60%	YES	YES	NO	NO	YES						
**IMCI-criteria for antibiotic treatment**			CNS	Hydration/ nutrition	Respiratory	Other							
	YES	100%	(YES)	(YES)	YES	(YES)							
% n/N			100.0%	100.0%	100.0%	100.0%							
			"drowsy", "lethargic", "very sick child"	"severe dehydration"		"swelling behind ear"							
			convulsion	"severe difficulty drinking"		ear pain							
			"meningismus"	"marasmus"		ear discharge <14							
				MUAC									
				weight for age <-3z score		bloody diarrhea							

	Prediction Rule	used for validation	% of predictors	variables used (original or proxy)				
	**iCCM-criteria for antibiotic treatment**			CNS	Hydration/ nutrition	Respiratory	Other	
		YES	100%	(YES)	(YES)	YES	(YES)	
% n/N				100.0%	100.0%	100.0%	100.0%	
				"drowsy", "lethargic", "very sick child"	"severe dehydration"		HIV positive	
					"severe difficulty drinking"		bloody diarrhea	
					"marasmus"			
				convulsion	MUAC			
					weight for age <-3z score			
	**ALMANACH-referral criteria or criteria for antibiotics present**			CNS	Hydration/ nutrition	Respiratory	Other	
		YES	100%	(YES)	(YES)	(YES)	(YES)	
% n/N				100.0%	100.0%	100.0%	100.0%	
				"drowsy", "lethargic", "very sick child"	"severe dehydration"	lower chest indrawing	jaundice	
					"severe difficulty drinking"	stridor	ear pain	
					"marasmus"	RR >50/min	ear discharge <14	
				convulsion			bloody diarrhea	
				"meningismus"			urine dipstick	
	**NICE traffic light system-amber or red present**			Colour	Activity	Respiratory	Circulation and	Other
			90%	(YES)	(YES)	YES	(YES)	(YES)
% n/N				100.0%	100.0%	100.0%	100.0%	100.0%
				"palmor pallor"	"drowsy", "lethargic", "very sick child"		"severe difficulty drinking"	axillary temperature
								"convulsion"
							heartrate	"meningismus"
								duration of fever
								"limb pain"

Prediction Rule	used for validation	% of predictors	variables used (original or proxy)			
**American Academy of Emergency Physicians Guidelines**			Ill appearing	Positive chest radiography (to be obtained if: T≥39°C (T>39 and WBC >20K/mm3)	Positive urine leuc + nitrite (to be obtained in male <1 year and female <2 year)	T≥39°C and WBC>15K/mm3
		100%	(YES)	(YES) **	YES	YES
% n/N			100.0%	21.0%	75.4%	99.8%
			"drowsy", "lethargic", "very sick child"			

*urine dipstick was only obtained in selected children per predefined algorithm, ** chest radiography was obtained for patients with cough and tachypnea, not based on temperature and WBC

C-reactive protein (CRP), procalcitonin (PCT), serious bacterial infection (SBI), white blood cell count (WBC), oxygen saturation (SaO2)

Prediction rules shaded in green could be included into the validation exercise, those shaded red could not be included. As for predictors, those in green font matched between the derivation and validation sets, for those with yellow font proxies were used, those with red font were not recorded in the validation dataset.
